# Module-based construction of plasmids for chromosomal integration of the fission yeast *Schizosaccharomyces pombe*

**DOI:** 10.1098/rsob.150054

**Published:** 2015-06-24

**Authors:** Yasutaka Kakui, Tomonari Sunaga, Kunio Arai, James Dodgson, Liang Ji, Attila Csikász-Nagy, Rafael Carazo-Salas, Masamitsu Sato

**Affiliations:** 1 Chromosome Segregation Laboratory, The Francis Crick Institute, Lincoln's Inn Fields Laboratories, 44 Lincoln's Inn Fields, London WC2A 3LY, UK; 2 Laboratory of Cytoskeletal Logistics, Department of Life Science and Medical Bioscience, Graduate School of Advanced Science and Engineering, Waseda University TWIns, 2-2 Wakamatsucho, Shinjuku, Tokyo 162-0056, Japan; 3 Department of Biophysics and Biochemistry, Graduate School of Science, University of Tokyo, 7-3-1 Hongo, Tokyo 113-0033, Japan; 4 The Gurdon Institute, University of Cambridge, Tennis Court Road, Cambridge CB2 1QN, UK; 5 Department of Genetics, University of Cambridge, Downing Street, Cambridge CB2 3EH, UK; 6 Department of Computational Biology, Research and Innovation Centre, Fondazione Edmund Mach, San Michele all’Adige 38010, Italy; 7 Randall Division of Cell and Molecular Biophysics and Institute for Mathematical and Molecular Biomedicine, King's College London, London SE1 1UL, UK

**Keywords:** fission yeast, chromosomal integration, plasmid, cloning, fluorescent protein

## Abstract

Integration of an external gene into a fission yeast chromosome is useful to investigate the effect of the gene product. An easy way to knock-in a gene construct is use of an integration plasmid, which can be targeted and inserted to a chromosome through homologous recombination. Despite the advantage of integration, construction of integration plasmids is energy- and time-consuming, because there is no systematic library of integration plasmids with various promoters, fluorescent protein tags, terminators and selection markers; therefore, researchers are often forced to make appropriate ones through multiple rounds of cloning procedures. Here, we establish materials and methods to easily construct integration plasmids. We introduce a convenient cloning system based on Golden Gate DNA shuffling, which enables the connection of multiple DNA fragments at once: any kind of promoters and terminators, the gene of interest, in combination with any fluorescent protein tag genes and any selection markers. Each of those DNA fragments, called a ‘module’, can be tandemly ligated in the order we desire in a single reaction, which yields a circular plasmid in a one-step manner. The resulting plasmids can be integrated through standard methods for transformation. Thus, these materials and methods help easy construction of knock-in strains, and this will further increase the value of fission yeast as a model organism.

## 1. Background

Recent advances of genetics and molecular biology of the fission yeast *Schizosaccharomyces pombe* by researchers have rendered it useful as a model organism to study many aspects of cellular phenomena.

In the long history of fission yeast studies, plasmids have been widely used to express external genes (including epitope-tagged *S. pombe* genes and genes from external species). Circular plasmids exist as extrachromosomal copies and are not integrated into chromosomes. Plasmid vectors normally contain a marker gene conferring amino acid autotrophy, so that transformants can be selected in selective media (such as EMM and SD) lacking the corresponding amino acid. The effect of expression from plasmids varies in individual cells and colonies, because the copy number of plasmids varies in individual cells. This situation may cause an inconsistency in statistical quantification of the phenotype induced by the expression from plasmids. We therefore need to pay special attention to compare the expression level among colonies when careful quantification is necessary.

This can be solved by integration of external genes into chromosomes, taking advantage of an intrinsic high activity of mitotic homologous recombination. This limits the copy number of the gene to one per cell, which is convenient for stable expression of external genes and quantitative evaluation of effects caused by the expression. Therefore, there are increasing demands on integration of external genes to be expressed in *S. pombe* cells. For instance, the TEV protease derived from tobacco etch virus [1] can be expressed in *S. pombe* cells, to cleave target proteins bearing the recognition sites [2]. Multiple tandem copies of GBP (GFP-binding protein) can be also introduced to *S. pombe* cells to induce oligomerization of GFP-fusion proteins [3].

**Figure 1.**
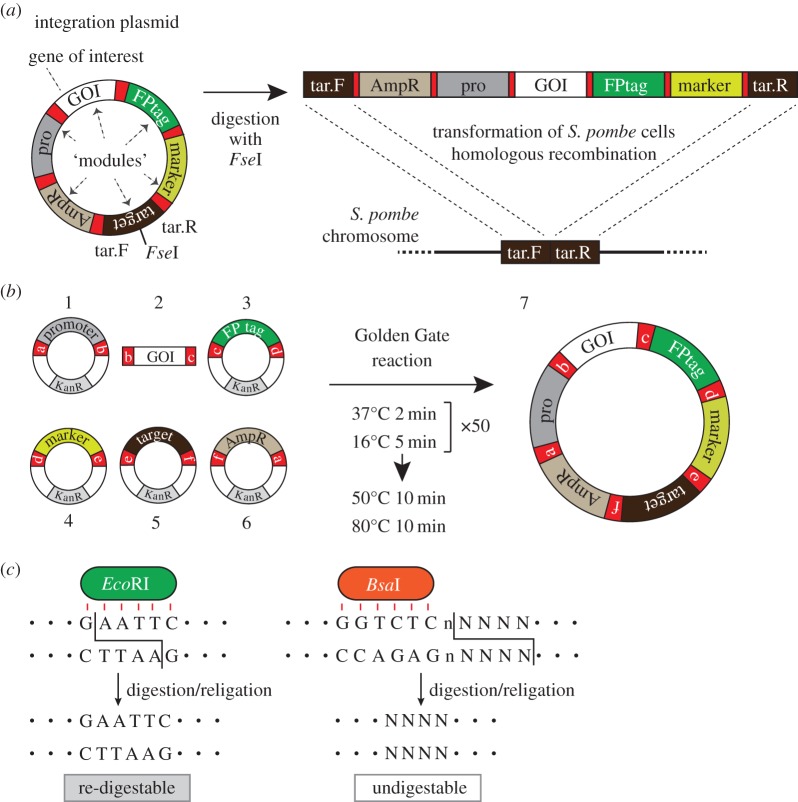
The Golden Gate method to create integration plasmids. (*a*) A schematic of an integration plasmid. (left) An example of an integration plasmid to express a GOI in fusion with a fluorescent protein (FP) tag under a promoter. A target module comprises tar.F and tar.R regions separated by an *Fse*I restriction site. (right) An integration plasmid can be linearized with *Fse*I, and tar.F and tar.R sequences are targeted to the homologous sequences on the *S. pombe* chromosome, to induce homologous recombination. (*b*) A schematic for the Golden Gate reaction. (left) Examples of module elements. Modules are given either as plasmids (1, 3–6) or as PCR products (2). Modules for a promoter module (1), GOI (2), an FPtag (3), a selection marker (4), a target region (5), the vector backbone (6). a–f: cohesive ends to connect modules 1–6 in this order. (right) A reaction protocol for the Golden Gate reaction by the mixture of 1–6 and the resulting circular integration plasmid (7). Each module plasmid (1, 3–6) contains the kanamycin resistance gene (KanR), whereas the final product (integration plasmid, 7) is ampicillin resistant. (*c*) Unique property of *Bsa*I. (left) *Eco*RI, a standard restriction enzyme, cleaves its recognition site, therefore digestion and religation can be repeated. (right) By contrast, *Bsa*I has separate sites for recognition (GGTCTC) and digestion (NNNN; any four bases).

For integration of external genes, we need to construct plasmids containing the following gene elements: a gene of interest (GOI), a promoter and a terminator to express the GOI, a selectable marker gene for colony selection, and sequences to target the entire construct to a certain part of chromosomes (see figure 1*a* for a schematic). We may also need any of fluorescent protein tag genes to fuse with the GOI. Those elements are termed ‘modules’ in this study.

All of those necessary modules need to be cloned in the proper order in a circular plasmid, which we call an ‘integration plasmid’ (figure 1*a*). It is then linearized and introduced into *S. pombe* cells so that homologous recombination takes place.

A number of plasmids for integration have been constructed and have greatly contributed to studies using *S. pombe*. Those include simple plasmids (pJK4, pJK148 and pJK210), which enable integration of a cloned gene fragment at the *leu1*^+^ or *ura4*^+^ locus [4]. More detailed plasmids were later developed: pDUAL and pDUAL2, in which *nmt* promoters (Pnmt1/Pnmt41/Pnmt81) have been cloned in advance to promote expression of a GOI [5]. Some of those plasmids also contain GFP or FLAG tags at the N- or C-terminus of the GOI. Later the authors used other gene loci (*arg1*, *his3* and *lys1*) as target sites of integration, and introduced three antibiotic resistance genes as a selection marker (*kan*/*hph*/*bsd*), as well as the *ura4*^+^ gene [6,7]. More recently, a number of integration plasmids have been introduced, providing a variety of options in N- and C-terminal tagging (3Pk, 3HA, HisMyc, EGFP and GST) [8].

Thus, development of those modules expanded the scope of use of integration plasmids. Users normally choose plasmids suitable for their own experiments from the catalogue of those ‘ready-made’ plasmids, and insert a GOI into them. From the current catalogue, however, it is often hard to find an optimal integration plasmid that suffices our demand for promoters, tags, selection markers and so on. This is because a growing number of new modules (such as new fluorescent proteins and selection marker genes) have been invented. Accordingly, the number of integration plasmids that we need to prepare surges, in order to fulfil the collection of integration plasmids, considering combination of many kinds of modules. To make such an integration plasmid, we may need to modify one of the existing plasmids by repeating DNA ligation and plasmid minipreps several times until all the DNA fragments are connected in the right order. It is therefore beneficial to build up materials and methods that enable us to create those constructs easily and systematically.

The Golden Gate shuffling method is a powerful method developed by Marillonnet and co-workers [9–11], which performs ligation of many DNA fragments in a single reaction. Using this method, we now can connect many necessary modules chosen from various options in a single reaction. This enables us to construct ‘custom-made’ integration plasmids with any combination of modules on demand in a single reaction, even when there is no existing ready-made integration plasmid that perfectly meets our demand. We therefore decided to apply this method to construction of integration plasmids.

## 2. Results

### 2.1. What we provide in this paper

Here, we present protocols for one-step plasmid construction, based on the Golden Gate DNA shuffling method. We also present a library of plasmids harbouring various modules. We call them ‘module plasmids’, which can be used as entry plasmids for the Golden Gate reaction. Modules in the library include a number of representative gene elements that are frequently used in *S. pombe* studies, such as P*nmt1*, P*nmt41* and P*nmt81* promoter modules, and kanMX, hphMX and natMX marker modules.

We also introduce new ‘target modules’ for gene targeting. A target module must be present in an integration plasmid to induce homologous recombination.

### 2.2. Outline of integration using integration plasmids

The outline of the integration method used in this study is as follows. For integration of a gene construct, an ‘integration plasmid’ as shown in figure 1*a* needs to be constructed. It needs to contain a number of gene modules that are necessary to achieve expression of a GOI and its integration. The example plasmid in figure 1*a* is to express a ‘GOI’ (module) in *S. pombe* cells. To visualize protein localization of the gene product, a fluorescent protein tag (an ‘FPtag’ module) is to be fused in frame at the C-terminal end of the GOI. The fusion gene construct is to be expressed under a promoter (a ‘pro’ module). To target the gene construct to a part of *S. pombe* chromosomes, the sequence of the target site (‘target’ module) needs to be cloned in the plasmid. A selection marker gene (a ‘marker’ module) is necessary for selection of proper *S. pombe* transformants. The ‘vector’ module contains a vector backbone including the ampicillin resistance gene (AmpR), which is useful to amplify integration plasmids using *E. coli*.

The integration plasmid needs to be linearized in the middle of the target module prior to *S. pombe* transformation. The target modules we provide in this study contain an *Fse*I restriction site in their middle. Digestion with *Fse*I splits the target module into two: tar.F and tar.R (figure 1*a*). When the fragment is introduced into yeast cells using standard *S. pombe* transformation methods (e.g. [12]), the linearized fragment can be integrated into the homologous target site in the *S. pombe* chromosomal DNA by the homologous recombination machinery (figure 1*a*).

### 2.3. Construction of integration plasmids using the Golden Gate method

To create integration plasmids, we applied the Golden Gate shuffling method (figure 1*b*). The method has been invented by Marillonnet and co-workers [9]. It uses only one restriction enzyme, *Bsa*I, for connection of many DNA fragments in a desired order.

To make the integration plasmid in figure 1*a*, for instance, each of six modules needs to be prepared in advance, either in a plasmid or in a fragment: a promoter module ((1), figure 1*b*), a GOI module (2), a FPtag module (3), a module for the selective marker gene ((4); this module also contains a terminator for the GOI but is omitted in figure 1*b* for simplicity), a target module to induce homologous recombination (5) and the vector module (6). All modules except (2) have been cloned in the pCR-Blunt II-TOPO vector, and the GOI module (2) has been amplified through PCR. Note that a *Bsa*I site flanks each end of all the modules (a–f, figure 1*b*). The restriction enzyme has the unique property that enables ordered connection of many restriction fragments using a single enzyme. This is explained as follows.

First, a standard restriction enzyme, *Eco*RI, recognizes and cleaves the sequence 5′-G!GATCC-3′ (! is the actual cutting site). The cohesive end created by *Eco*RI is therefore unique (figure 1*c*). *Bsa*I, on the other hand, specifically recognizes 5′-GGTCTC-3′. Notably, the enzyme cleaves the sequence right after it: the sequence for recognition and digestion is therefore 5′-GGTCTCn!NNNN-3′, in which ‘n’ and ‘N’ represent any nucleotide (figure 1*c*). *Bsa*I thus creates DNA fragments with a variety of overhang sequences (cohesive end; NNNN), but their religation is limited only to among those two with the same overhang sequence. By modulating the sequences, we can selectively connect those fragments in an order we desire.

**Figure 2.**
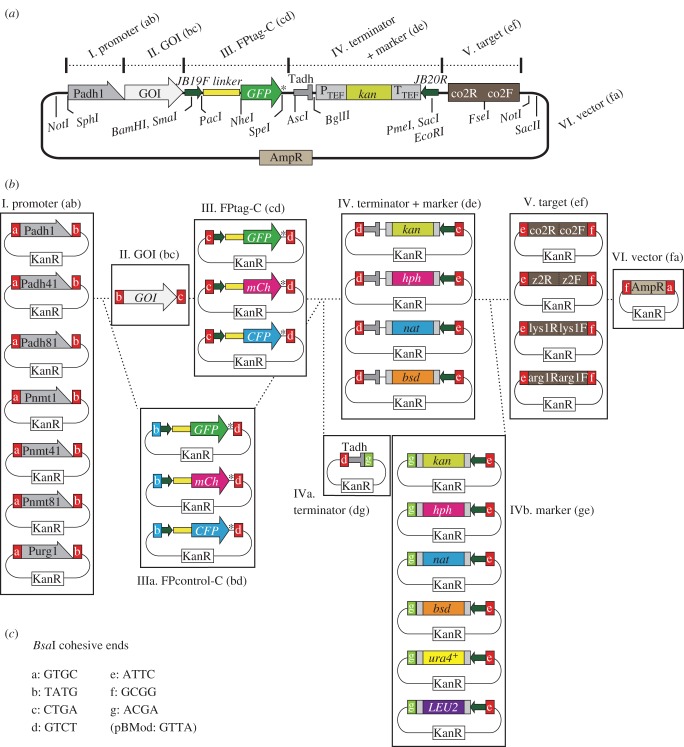
Choice of module plasmids for expression of a C-terminal tagged GOI. (*a*) Detailed illustration of an integration plasmid for expression of the GOI–GFP fusion (C-terminal GFP tag). Modules I–V are connected in the pFA6a-based vector (module VI) in that order. In this example, the *adh1* promoter (selected from group I modules) drives expression of the fusion gene of the GOI (GOI (bc), II) with GFP (FPtag-C (cd), III). T*adh* serves as a terminator. *kan* (P_TEF_, promoter; T_TEF_, terminator) is a selection marker used after *S. pombe* transformation (module IV). Target module (V) is the sequence that is targeted to a homologous sequence in *S. pombe* chromosomes. Useful restriction sites are also indicated. Digestion with *Not*I separates the vector and other modules. JB19F and JB20R correspond to sequences commonly used in PCR-based gene targeting [12]. a–f in module names indicate the names of *Bsa*I cohesive ends used therein (*c*). AmpR, the ampicillin resistance gene. (*b*,*c*) List of module plasmids created in this study. (*b*) Modules are categorized as groups I–VI in boxes. Choose one module from each group to mix. II. The GOI (bc) is made through PCR to add cohesive ends (‘b’ and ‘c’). Group IIIa, instead of II and III, can be used to make control strains. Modules for the *adh* terminator and a selection marker gene can be supplied together (group IV). Alternatively, each module can be chosen separately: a terminator (group IVa) and a selection marker (group IVb). (*c*) Sequences of cohesive ends named a–g. Note that the module vector pBMod contains a *Bsa*I site with the cohesive end GTTA.

These cohesive ends with various overhang sequences are named as ‘a’, ‘b’, etc. For instance, a promoter module has two *Bsa*I sites with ‘a’ and ‘b’ (1, figure 1*b*; see figure 2*c* for sequence), as these two sites are designed to have distinct overhang sequences that are specific to the sites. Next, the GOI module (2) is flanked by two distinct sequences ‘b’ and ‘c’, in which b is same as the overhang b in the module (1).

After digestion with *Bsa*I, cohesive ends a, b, c, etc. are generated and exposed, but only DNA fragments that contain the same overhang can be ligated efficiently. For instance, the b site of (1) can be connected with the b site in the GOI fragment (2). Similarly, c in (2) can be exclusively ligated with c in (3). Moreover, digestion with *Bsa*I results in loss of its recognition sequence (5′-GGTCTC-3′) at one end. Once ligation between two fragments without the recognition sequence is made, the site is no longer re-digestible (figure 1*c*). Using this unique property, we can connect many DNA fragments in a selective manner, with only a single enzyme *Bsa*I. In the example, these six modules can be connected in the order we designed, which yields the integration plasmid (7, figure 1*b*).

The experimental procedure for the Golden Gate reaction is simple. Prepare all of (1)–(6) and then mix them in a tube. *Bsa*I and DNA ligase is subsequently added to the mixture, to repeat cutting and ligation by the thermal cycler (figure 1*b*; see below for details). The sample is then introduced into *E. coli* and correct transformants are selected with ampicillin-containing plates, as in standard methods of cloning.

### 2.4. What you need to prepare when making an integration plasmid

**Figure 3.**
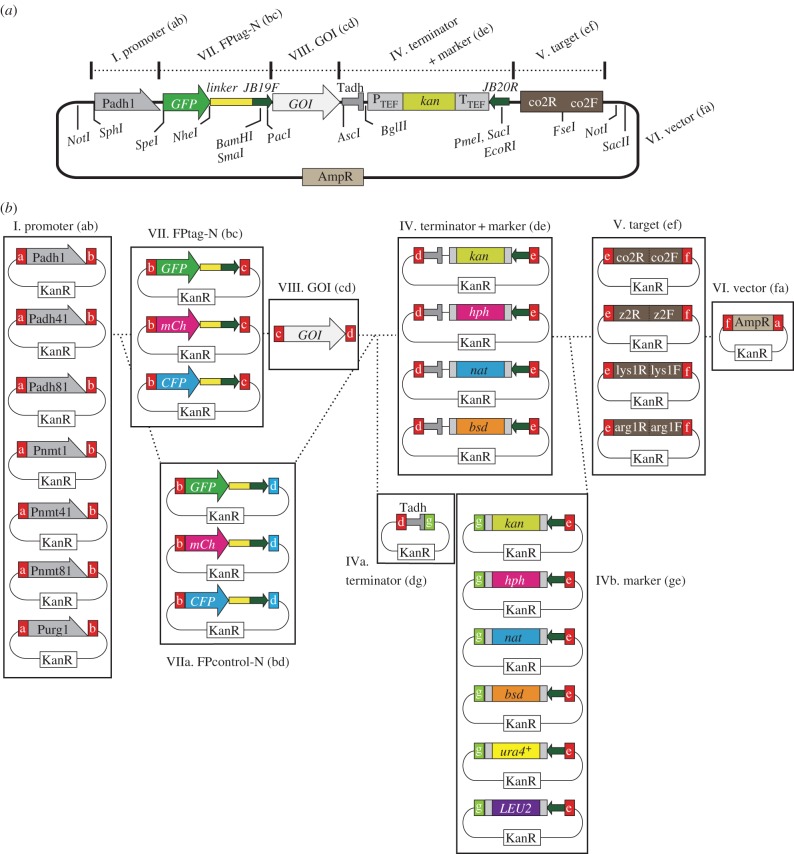
Choice of module plasmids for expression of FP-GOI. (*a*) Detailed construct of an integration plasmid for expression of a GFP–GOI (N-terminal GFP tag). Modules chosen from groups I, VII, VIII, IV, V and VI are connected in this order. The *adh1* promoter (P*adh1*, group I) drives expression of the fusion gene GFP–GOI (groups VII and VIII). The plasmid digested with *Fse*I can be targeted to the co2 locus of chromosome I by the co2 module (IV). Representative restriction sites are also shown. (*b*) List of module plasmids used for Golden Gate reaction to construct integration plasmids to express a GOI with an N-terminal FP tag (also see table 1). The GOI (cd) is a PCR product containing cohesive ends (‘c’ and ‘d’). To make control plasmids expressing fluorescent proteins without a GOI, a module from group VIIa can be used instead of VII and VIII. See figure 2*b* for other module groups. a–g: cohesive ends, sequences of which are shown in figure 2*c*.

Here we created a number of common modules cloned in plasmids so that researchers can share those frequently used modules. All the modules we created (except for the GOI) were cloned into the plasmid vector pCR-Blunt II-TOPO (Invitrogen), which confers kanamycin resistance to *E. coli*, and named pBMod in this study. All module plasmids we created are systematically numbered and listed in table 1, and are illustrated in figures 2 and 3.

**Table 1.** Module plasmids created in this study. Systematic numbers (denoted with # throughout the manuscript) and plasmid names are listed with module contents. Each module was PCR-amplified from the indicated origin template to add particular cohesive ends, the names of which are listed in ‘names of cohesive ends’ (a–g shown in figure 2*c* and table 2). The amplified module fragment was cloned into pCR-Blunt II-TOPO. The vector name pBMod indicates pCR-Blunt II-TOPO-based module plasmid. An Excel version of this table is available in the electronic supplementary material.

	module plasmids		modules (contents)	name of cohesive ends (left, right)	origin	literature	notes
	number	name					
	I. Promoter (ab)						
figures 2 and 3	1	pBMod-Padh1(ab)	Padh1	a, b	*S. pombe* genome	Russell & Hall [13]	
	2	pBMod-Padh41(ab)	Padh41	a, b	Padh1, mutagenesis	Yamagishi *et al*. [14]	
	3	pBMod-Padh81(ab)	Padh81	a, b	Padh1, mutagenesis	Yokobayashi & Watanabe [2]	
	4	pBMod-Pnmt1(ab)	Pnmt1	a, b	*S. pombe* genome	Maundrell [15]	
	5	pBMod-Pnmt41(ab)	Pnmt41	a, b	Pnmt1, mutagenesis	Basi *et al*. [16]	
	6	pBMod-Pnmt81(ab)	Pnmt81	a, b	Pnmt1, mutagenesis	Basi *et al.* [16]	
	7	pBMod-Purg1(ab)	Purg1	a, b	pFA6a-kanMX6-Purg1	Watt *et al*. [17]	
	III. FPtag-C (cd)						
figure 2	8	pBMod-L-GFP(cd)	L-GFP^a^	c, d	pFA6a-GFP(S65T)-kanMX	Bähler *et al.* [12]	BsaI removed
(C tag)	9	pBMod-L-mCherry(cd)	L-mCherry^a^	c, d	pFA6a-mCherry-hphMX	Sato *et al.* [18]	
	10	pBMod-L-ECFP(cd)	L-ECFP^a^	c, d	pFA6a-ECFP-natMX	Sato *et al.* [18]	
	IIIa. FPcontrol-C (bd)						
	11	pBMod-cont-L-GFP(bd)	control L-GFP^a^	b, d	pFA6a-GFP(S65T)-kanMX	Bähler *et al.* [12]	BsaI removed
	12	pBMod-cont-L-mCherry(bd)	control L-mCherry^a^	b, d	pFA6a-mCherry-hphMX	Sato *et al.* [18]	
	13	pBMod-cont-L-ECFP(bd)	control L-ECFP^a^	b, d	pFA6a-ECFP-natMX	Sato *et al.* [18]	
figure 3	VII. FPtag-N (bc)						
(N tag)	14	pBMod-GFP-L(bc)	GFP-L^a^	b, c	pFA6a-GFP(S65T)-kanMX	Bähler *et al.* [12]	BsaI removed
	15	pBMod-mCherry-L(bc)	mCherry-L^a^	b, c	pFA6a-mCherry-hphMX	Sato *et al.* [18]	
	16	pBMod-ECFP-L(bc)	ECFP-L^a^	b, c	pFA6a-ECFP-natMX	Sato *et al.* [18]	
	VIIa. FPcontrol-N (bd)						
	17	pBMod-cont-GFP-L(bd)	control GFP-L^a^	b, d	pFA6a-GFP(S65T)-kanMX	Bähler *et al.* [12]	BsaI removed
	18	pBMod-cont-mCherry-L(bd)	control mCherry-L^a^	b, d	pFA6a-mCherry-hphMX	Sato *et al.* [18]	
	19	pBMod-cont-ECFP-L(bd)	control ECFP-L^a^	b, d	pFA6a-ECFP-natMX	Sato *et al.* [18]	
figures 2 and 3	IV. Terminator + Marker (de)						
	20	pBMod-Tadh-kan(de)	Tadh-*kan*	d, e	pFA6a-GFP(S65 T)-kanMX	Bähler *et al.* [12]	
	21	pBMod-Tadh-hph(de)	Tadh-*hph*	d, e	pFA6a-mCherry-hphMX	Sato *et al.* [18]	
	22	pBMod-Tadh-nat(de)	Tadh-*nat*	d, e	pFA6a-ECFP-natMX	Sato *et al.* [18]	
	23	pBMod-Tadh-bsd(de)	Tadh-*bsd*	d, e	pCR2.1-bsd	Kimura *et al.* [19]	
	IVa. Terminator (dg)						
	24	pBMod-Tadh(dg)	Tadh	d, g	pFA6a-GFP(S65 T)-kanMX	Bähler *et al.* [12]	
	IVb. Marker (ge)						
	25	pBMod-kan(ge)	*kan*	g, e	pFA6a-GFP(S65 T)-kanMX	Bähler *et al.* [12]	
	26	pBMod-hph(ge)	*hph*	g, e	pFA6a-mCherry-hphMX	Sato *et al.* [18]	
	27	pBMod-nat(ge)	*nat*	g, e	pFA6a-ECFP-natMX	Sato *et al.* [18]	
	28	pBMod-bsd(ge)	*bsd*	g, e	pCR2.1-bsd	Kimura *et al.* [19]	
	29	pBMod-ura4 + (ge)	*ura4*+	g, e	pREP2	Maundrell [15]	
	30	pBMod-LEU2(ge)	*LEU2*	g, e	pREP1	Maundrell [15]	
figures 2 and 3	V. Target (ef)						
	31	pBMod-co2(ef)	co2	e, f	*S. pombe* genome	this study	
	32	pBMod-Z2(ef)	Z2	e, f	*S. pombe* genome	Akera *et al.* [20]	
	33	pBMod-lys1(ef)	*lys1*	e, f	*S. pombe* genome	pombase.org	
	34	pBMod-arg1(ef)	*arg1*	e, f	*S. pombe* genome	pombase.org	
figures 2 and 3	VI. Vector (fa)						
	35	pBMod-AmpVec(fa)	*AmpR* vector	f, a	pFA6a	Wach *et al.* [21]	*Bsa*I removed, lacking a 137 bp fragment (see Material and methods for details)

^a^L, linker.

In many cases, therefore, the only material you need to prepare by yourselves is the GOI module (2). This can be amplified from a template sequence (such as genome or cDNA) using a pair of oligonucleotide primers, both of which contain a *Bsa*I site (the recognition site with a cohesive end) at the 5′ end, in order to flank the GOI with two distinct cohesive ends. Cloning of modules into vectors is not necessarily required.

Also, researchers may want to use other modules that are not yet cloned in this study (e.g. new promoters, fluorescent proteins and selection markers). Those new modules can be prepared similarly, either as plasmids or as fragments. The detailed instructions will be described below.

### 2.5. Materials

Figure 2*a* illustrates construction of a typical integration plasmid to express a GOI fused with an FP protein in frame at its C-terminus (GOI-FP). It consists of six modules (I–VI, figure 2*a*) connected in this order.

Here, modules are classified into groups according to their functions. For example, the group ‘I. Promoter (ab)’ (figure 2*a*,*b*) is the group for promoter modules, where ‘(ab)’ indicates names of two cohesive ends (‘a’ and ‘b’, the left one is at the 5′ side) generated by *Bsa*I digestion (figure 2*b,c* and table 1). The numbers with ‘#’ throughout the manuscript denote systematic numbers for each module plasmid. For example, the module plasmid ‘#1 in the group I. Promoter (ab)’ indicates that this plasmid contains the gene construct ‘a-Padh1-b’ cloned in the vector pBMod.

In the example of figure 2*a*, the fusion gene of GOI (group II. GOI (bc)) and GFP at the C-terminus (#8, group III. FPtag-C (cd)) via a linker sequence is placed under the *adh1* promoter (Padh1, #1, group I. Promoter (ab)) and the terminator (Tadh, a part of modules in group IV. Terminator + Marker (de)). To target the gene construct to *S. pombe* chromosomes, a sequence from a region on the chromosome I (termed co2, #31, group V. Target (ef)) is used. For selection of transformants after integration, an antibiotic selection marker gene, *kan*, is used (Ptef-*kan*-Ttef, a part of #25, group IV). The vector backbone (#35, group VI. Vector (fa)) is derived from pFA6a, which has been commonly used as a PCR template for standard methods for gene targeting [12,22–25].

### 2.6. Modules for FP tagging at C-terminus of GOI (GOI–FP)

Figure 2*b* illustrates a catalogue of modules we present in this study (except for the GOI).

I. *Promoter* (*ab*) *modules*

We have chosen seven promoters that are commonly used in fission yeast studies: Pnmt1 (#1), Pnmt41 (#2), Pnmt81 (#3), Padh1 (#4), Padh41 (#5), Padh81 (#6) and Purg1 (#7). Those include a series of the *nmt* promoter (Pnmt1, Pnmt41 and Pnmt81), widely used as artificial promoters to control expression of external genes. Briefly, those were derived from the promoter of the endogenous *nmt1* gene [15]. Pnmt41 has weaker promoter activity than Pnmt1, and Pnmt81 is even weaker [16]. The transcription level from those promoters can be controlled by addition or removal of thiamine in the medium. The expression is repressed in the presence of thiamine, whereas it is induced in its absence. Pnmt1 is often used to overexpress the downstream genes (e.g. pREP1 plasmid [26]).

A series of the *adh1* promoter variants (Padh1, Padh41 and Padh81): Padh1 is derived from the promoter region of the endogenous *adh1* gene, whereas Padh41 and Padh81 are mutant versions of Padh1 with weaker promoter activities [2,27]. The *urg1* promoter (Purg1) is an inducible promoter, which is activated in response to uracil [17].

As we mentioned earlier, it is possible to make new modules for other promoters (e.g. promoters of your interest and newly introduced artificial promoters) [6,28,29]. A brief introduction to preparing such modules will be given below (table 2).

**Table 2.** Guideline for designing oligonucleotide primers with cohesive ends. When a new module is to be made, design a pair of oligonucleotide primers, the sequences of which are shown in the table. ‘Specific 20b’ corresponds to the 20-base sequence of the template gene to be amplified. The length (20 bases) can be varied depending on the situation. Note that the *Bsa*I recognition site is ‘GGTCTC’. By setting up the rule above to choose cohesive ends, we can modulate the order in which modules are connected. FWD, forward primer; REV, reverse primer; rev. comp., reverse complement. An Excel version of this table is available in the electronic supplementary material.

		cohesive ends				
	modules and oligo direction	name	sequences (rev. comp.)	sequences of oligonucleotide primers (bold indicates a cohesive end)	notes	plasmids using this end (see table 1 for numbers)
figures 2 and 3	I. Promoter (ab)					
	FWD	a	**GTGC**	5′-tttGGTCTCa**GTGC**-(specific 20b)-3′		# 1–7
	REV	b	**TATG (CATA)**	5′-tttGGTCTCa**CAT****A**-(specific 20b)-3′	the cohesive end includes the initiation codon ATG for GOI	# 1–7
figure 2	II. GOI (bc)					
(C tag)	FWD	b	**TATG**	5′-tttGGTCTCa**T****ATG**-(specific 20b)-3′	the cohesive end includes the initiation codon ATG for GOI	
	REV	c	**CTGA (TCAG)**	5′-tttGGTCTCa**TCAG**-(specific 20b)-3′		
	III. FPtag-C (cd)					
	FWD	c	**CTGA**	5′-tttGGTCTCa**CTGA**aa-(specific 20b)-3′	aa is inserted to avoid a frame shift (CTG Aaa…)^a^	# 8–10
	REV	d	**GTCT (AGAC)**	5′-tttGGTCTCa**AGAC**tta-(specific 20b)-3′	tta serves as a termination codon for FP	# 8–10
	IIIa. FPcontrol-C (bd)					
	FWD	b	**TATG**	5′-tttGGTCTCa**T****ATG**-(specific 20b)-3′	the cohesive end includes the initiation codon ATG for FP	# 11–13
	REV	d	**GTCT (AGAC)**	same as II-REV(d)	tta serves as a termination codon for FP	# 11–13
	IIIa. GOI with small tag-C (bd)					
	FWD	b	**TATG**	5′-tttGGTCTCa**T****ATG**-(specific 20b)-3′	the cohesive end includes the initiation codon ATG for GOI	
	REV	d	**GTCT (AGAC)**	5′-tttGGTCTCa**AGAC**tta-***tag sequence***-(specific 20b)-3′	Tag sequence (e.g. HA, FLAG) is inserted before termination codon	
figures 2 and 3 (no tag)	IIIa. GOI (bd) (without tag)					
	FWD	b	**TATG**	5′-tttGGTCTCa**T****ATG**-(specific 20b)-3′	the cohesive end includes the initiation codon ATG for GOI	
	REV	d	**GTCT (AGAC)**	5′-tttGGTCTCa**AGAC**tta-(specific 20b)-3′	tta serves as a termination codon for GOI	
figure 3	VII. FPtag-N (bc)					
(N tag)	FWD	b	**TATG**	5′-tttGGTCTCa**T****ATG**-(specific 20b)-3′	the cohesive end includes the initiation codon ATG for FP	# 14–16
	REV	c	**CTGA (TCAG)**	5′-tttGGTCTCa**TCAG**-(specific 20b)-3′		# 14–16
	VIIa. FPcontrol-N (bd)					
	FWD	b	**TATG**	same as IV-FWD(b)	the cohesive end includes the initiation codon ATG for FP	# 17–19
	REV	d	**GTCT (AGAC)**	5′-tttGGTCTCa**AGAC**tta-(specific 20b)-3′	tta serves as a termination codon for FP	# 17–19
	VIIa. GOI with small tag-N (bd)					
	FWD	b	**TATG**	5′-tttGGTCTCa**T****ATG**-***tag sequence***-(specific 20b)-3′	Tag sequence is inserted between initiation codon and GOI	
	REV	d	**GTCT (AGAC)**	5′-tttGGTCTCa**AGAC**tta-(specific 20b)-3′	tta serves as a termination codon for GOI	
	VIII. GOI (cd)					
	FWD	c	**CTGA**	5′-tttGGTCTCa**CTG****A**tg-(specific 20b)-3′	start GOI from the second codon in frame after Atg for GOI	
	REV	d	**GTCT (AGAC)**	5′-tttGGTCTCa**AGAC**tta-(specific 20b)-3′	tta serves as a termination codon for GOI	
figures 2 and 3	IV. Terminator + Marker (de)					
	FWD	d	**GTCT**	5′-tttGGTCTCa**GTCT**-(specific 20b)-3′		# 20–23
	REV	e	**ATTC (GAAT)**	5′-tttGGTCTCa**GAAT**-(specific 20b)-3′		# 20–23
	IVa. Terminator (dg)					
	FWD	d	**GTCT**	5′-tttGGTCTCa**GTCT**-(specific 20b)-3′		# 24
	REV	g	**ACGA (TCGT)**	5′-tttGGTCTCa**TCGT**-(specific 20b)-3′		# 24
	IVb. Marker (ge)					
	FWD	g	**ACGA**	5′-tttGGTCTCa**ACGA**-(specific 20b)-3′		# 25–30
	REV	e	**ATTC (GAAT)**	5′-tttGGTCTCa**GAAT**-(specific 20b)-3′		# 25–30
figures 2 and 3	V. Target (ef)					
	FWD	e	**ATTC**	5′-tttGGTCTCa**ATTC**-(specific 20b)-3′		# 31–34
	REV	f	**GCGG (CCGC)**	5′-tttGGTCTCa**CCGC**-(specific 20b)-3′		# 31–34
figures 2 and 3	VI. Vector (fa)					
	FWD	f	**GCGG**	5′-tttGGTCTCa**GCGG**aaGCGGCCGC-(specific 20b)-3′	includes the *Not*I site (GCGGCCGC)	# 35
	REV	a	**GTGC (GCAC)**	5′-tttGGTCTCa**GCAC**GCGGCCGC-(specific 20b)-3′	includes the *Not*I site (GCGGCCGC) aa is inserted to avoid an overlap with the BsaI site	# 35

^a^The codon frame is indicated with a space.

II. *GOI* (*bc*) *module: for C-terminal tagging*

This module is supposed to vary depending on individual researchers. How to prepare your own modules for your GOI will be described below (table 2). When an FP tag is to be fused at the N-terminus of GOI, see figure 3. Alternatively, if you use no FP tags, see §2.6.IIIa.

III. *FPtag-C* (*cd*)*: fluorescent protein modules for C-terminal tagging of GOI* (*bc*)

The C-terminus of the GOI (bc) can be tagged with one of the FPtag-C (cd) modules listed in III (figure 2*b* and table 1).

We made modules for GFP (#8), mCherry (#9) and ECFP (#10). Each FPtag module has a linker sequence with the JB19F sequence (figure 2*a*,*b*). The JB19F sequence consists of 19 bases, which lacks the first base of the 20-bp Fwd sequence commonly used in pFA6a-based plasmids [12]. The actual sequence of the JB19F and the linker is: 5′-aGGATCCCCGGGTTAATTAAgGGAGCAGGTGCTGGTGCTGGTGCTGGAGCATTTTCCGTCCCCATTACAACAGCTAGC-3′ (underlined, JB19F). The translated amino acid sequence is RIPGLIKGAGAGAGAGAFSVPITTAS.

IIIa*. FPcontrol-C* (*bd*)*: control constructs without GOI (bc)*

We may need to construct control strains at the same time, which has the FPtag module without the GOI. In this case, control FPtag modules (listed in figure 2*b* and table 1) can be used instead of modules II and III. Each control FPtag module contains an FPtag flanked by cohesive ends ‘b’ and ‘d’, so that the control module can be connected directly to the promoter module (I) and the selection marker module (IV) (figure 2*b*).

When we want to express a GOI without a fluorescent protein tag, such GOI fragments should be prepared with cohesive ends ‘b’ and ‘d’ at the 5′- and 3′-ends, respectively. This enables direct connection of the GOI with the promoter and the terminator modules, by skipping requirement of an FPtag module. Although we have no module plasmids for other small tags (e.g. FLAG and HA), we can achieve such tagging by including the tag sequence into an oligonucleotide primer to amplify the GOI. We present sequences of the primers for C-terminal FLAG and HA tagging. All information for designing oligonucleotides is summarized in table 2. Alternatively, those tags may be prepared through oligonucleotide annealing (see §2.9.2 for details).

Thus, by modifying sequences of the cohesive ends, we can customize which modules to include or to exclude.

IV, IVa and IVb. *Terminator and selection marker modules*

In order to terminate transcription of GOI, a module containing a terminator is required. In general, researchers tend to use the terminator of the *adh1* gene (Tadh1) to express external genes [26]. Next, selection markers are necessary to select positive colonies that underwent correct transformation and homologous recombination. We have prepared *kan*, *hph*, *nat* and *bsd*, which confer resistance to the antibiotics geneticin (G418), hygromycin B, clonNAT and blasticidin S, respectively [12,19,24,25].

As we place a selection marker gene normally right after the terminator for the GOI, we prepared modules which contain both Tadh1 and a selection marker tandemly aligned; for example, Tadh1 + kan (#20), Tadh1 + hph (#21), Tadh1 + nat (#22) and Tadh1 + bsd (#23) in the group IV. terminator + marker (de) (figure 2*b* and table 1). These are flanked by cohesive ends ‘d’ and ‘e’ (figure 2*b*).

Alternatively, researchers may want to use another terminator instead of Tadh1. In this case, we recommend separating the terminator module (IVa. Terminator (dg)) and the selection marker modules (IVb. Marker (ge)) (figure 2*b*). The terminator module (#24, IVa) is flanked by cohesive ends ‘d’ and ‘g’. In this study, we present only Tadh1 cloned in pCR-Blunt II-TOPO. If you need another terminator, simply prepare the DNA fragment through PCR, with additional cohesive ends ‘d’ and ‘g’ (see below and table 2 for oligonucleotide design).

Selection marker modules without a terminator (IVb. Marker (ge)) are flanked by cohesive ends ‘g’ and ‘e’. In this study, we present six selection marker modules without a terminator for GOI: *kan* (#25), *hph* (#26), *nat* (#27) and *bsd* (#28), as well as *ura4*^+^ (#29) and *LEU2* (#30) (confers uracil and leucine autotrophy, respectively).

V. *Target* (*ef*) *modules*

**Figure 4.**
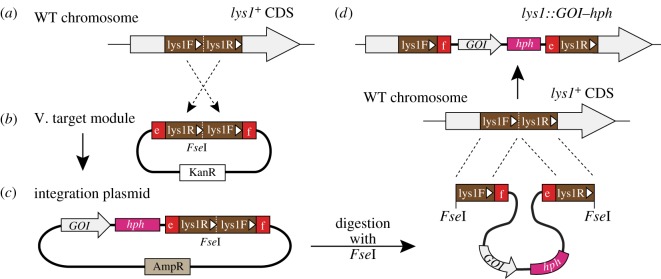
Construct of target modules. The *lys1* target module is shown as an example. (*a*,*b*) A schematic illustrating how target modules were made. (*a*) An approximately 1 kb region of the *lys1*^+^ gene was chosen as a target site. The first half of the region was termed lys1F, and the second half lys1R. White arrow and arrowheads indicate the direction of the coding sequence of *lys1*^+^. (*b*) In the *lys1* target module plasmid (#33, group V), the lys1R and lys1F fragments have been connected in this order with an *Fse*I site between them. e,f: cohesive ends shown in figure 2*c*. (*c*,*d*) The principle of how target module sequences work. (*c*) A schematic of an integration plasmid made with the module plasmid through a Golden Gate reaction. (*d*) Linearization of the plasmid with *Fse*I, followed by integration of the fragment into the *lys1*^+^ gene on *S. pombe* chromosome I through homologous recombination. The *lys1* gene of the resultant strain is disrupted (top).

Target modules contain a sequence used to induce homologous recombination in *S. pombe* cells. The ‘co2’ (#31) sequence in figure 2*a* is derived from a region on the *S. pombe* chromosome I (see Material and methods for details). Similarly, ‘z2’ (#32) is from a region on the chromosome II. ‘*lys1*’ (#33) and ‘*arg1*’ (#34) are derived from the endogenous *lys1* (chromosome I) and *arg1* (chromosome III) genes, respectively. How these modules were designed is described later (figure 4).

The restriction enzyme site *Fse*I is placed in the middle of target modules. Digestion of an integration plasmid with *Fse*I linearizes it, which is then efficiently targeted to its homologous sequence *in vivo* to induce recombination.

Make sure that the *Fse*I site is unique in the integration plasmid, in order to avoid multiple fragmentation of the integration plasmid after digestion. To minimize the risk, we intentionally chose the 8-base recognition enzyme *Fse*I for the purpose.

VI. *Vector* (*fa*) *module*

All modules above can be connected to the pFA6a-based vector module (#35, VI. Vector (fa), figure 2*b*) [21]. Note that this module is not exactly identical to the sequence of the original pFA6a. First, it is shorter than pFA6a. Second, the ampicillin resistance gene (AmpR) has a silent mutation. These were done in order to remove two internal *Bsa*I recognition sites in pFA6a (see Material and methods).

When designing an integration plasmid, make sure that all modules of your choice can be properly ligated in the desired order. Alphabetical names of the cohesive ends need to circularize, starting and ending with ‘a’: pBMod-Padh1 (ab) + GOI (bc) + pBMod-GFP (cd) + pBMod-Tadh-kan (de) + pBMod-co2 (ef) + pBMod-AmpVec (fa).

### 2.7. Modules for FP tagging at N-terminus of GOI (FP–GOI)

Expression of an N-terminally tagged protein (GOI) can be achieved in a similar way. Figure 3*a* illustrates the overview of an example of GOI fused with GFP at the N-terminal end (FP-GOI), whose expression is regulated by the *adh1* promoter and terminator.

Here, all modules for C-terminal tagging introduced in figure 2 can be used, except for II. GOI (bc) and III. FPtag-C (cd) modules. Namely, promoter modules (I), terminator + selection marker modules (IV, IVa and IVb), target modules (V) and the vector module (VI) can be shared for both N-terminal and C-terminal tagging. We need to use, however, modules that were particularly designed for N-terminal tagging: VII. FPtag-N (bc); VIIa. FPcontrol-N (bd) and the GOI with small tag (FLAG/HA)-N (bd); and VIII. GOI (cd), as described below (figure 3*a*,*b*; tables 1 and 2). Make sure that all cohesive ends you will use can be connected in the circular manner (e.g. a-b-c-d-e-f-a).

VII. *FPtag-N* (*bc*) *modules*

The second module for N-terminal tagging should be an FPtag (bc) module. It has a fluorescent protein gene followed by the linker sequence, which is flanked by cohesive ends ‘b’ and ‘c’ at the 5′- and 3′-ends, respectively (figure 3b).

VIIa. *FPcontrol-N* (*bd*) *modules and GOI with FLAG/HA tag-N* (*bd*) *modules*

As a control, another strain expressing an FPtag without the GOI may need to be constructed. For that purpose, modules belonging to group VIIa can be used (figure 3*b*). Modules for N-terminus FLAG/HA epitope tagging can be also prepared through PCR with an oligonucleotide primer containing the sequence of FLAG or HA epitope. Alternatively, such small epitope tags may be prepared through an oligonucleotide annealing method (see 2.9.2 for details). Similar to modules in the group IIIa, each module of the group contains an FPtag flanked by cohesive ends ‘b’ and ‘d’. See table 2 to design your oligonucleotide primers.

VIII. *GOI* (*cd*) *modules*

The third module is a GOI (cd) module. This should be prepared through PCR, with adding cohesive ends ‘c’ and ‘d’ at the 5′- and 3′-ends, respectively (figure 3*b*).

### 2.8. A practical protocol for the Golden Gate reaction

A concrete protocol for the Golden Gate reaction [9] is as follows. For efficient reaction, we use T4 DNA ligase (HC) from Promega (catalogue #M1794) and *Bsa*I (not HF) from New England Biolab (#R0535S). Similar enzymes by other suppliers may also work. We strongly recommend diluting each module element (including linear modules such as the GOI) in 20 fmol µl^−1^ (13.3 ng µl^−1^ of a 1-kilobase DNA; note that the vector pBMod is 3.5 kilobase). The concentration appears critical for efficient ligation. One microlitre (20 fmol) of each module is taken and mixed in a PCR tube. Fill up to 16 µl with ddH_2_O.

Add 2 µl of 10× buffer for ligase followed by 0.5 µl of T4 DNA ligase and 1.5 µl of *Bsa*I. The total volume will be 20 µl. The tube can be placed on a thermal cycler to perform all the reaction steps automatically. The Golden Gate reaction comprises basically two major repetitive steps followed by the termination reaction (figure 1*b*). The first step is DNA digestion by *Bsa*I (37°C, 2 min), and the second step is ligation (16°C, 5 min). Repeat these steps up to 50 times in total. Conditions such as temperature and duration of each step may need to be varied for optimization according to instructions of suppliers of enzymes used therein. Then shift up to 50°C for 10 min followed by the heat inactivation step (80°C, 10 min). The sample can be kept at 4–15°C.

The sample can be directly used for *E. coli* transformation. We normally use 2 µl of the reaction sample for transformation. Colonies on LB + Amp plates could be subjected to minipreps. Note that pCR-Blunt II-TOPO used as an entry vector carries the kanamycin resistance gene, so that unreacted entry vectors never grow on LB + Amp. This eliminates contamination of colonies with unreacted module plasmids.

Successfully ligated clones survive and accumulate during 50 cycles of repetitive *Bsa*I digestion and ligation, because once two cohesive ends without *Bsa*I recognition sites are connected, the site cannot be redigested (figure 1*a*). Correct clones must have all the modules connected in the right order we expect, and cloned in the AmpR-bearing vector (7, figure 1*b*). This can be confirmed by digestion with *Not*I (figures 2*a* and 3*a*).

A successful plasmid is then subjected to transformation of *S. pombe*. First, the plasmid is linearized with *Fse*I (figure 1*a*). Appropriate host strains of *S. pombe* are to be used for transformation with the linearized DNA, following a standard protocol using lithium acetate [12]. Homologous recombination takes place using the target module (figure 1*c*). After transformation, choose stable colonies that can grow on the solid medium containing appropriate antibiotics, or minimal medium without an appropriate supplement (leucine or uracil), depending on the marker gene you use.

Correct integration of the DNA fragment into the target site can be verified in the following ways. When either *lys1* or *arg1* target module is used, correct transformants should confer both antibiotics resistance and a lysine or arginine auxotroph, respectively. This can be tested with minimal media lacking lysine or arginine, respectively. When co2 and z2 are used, colony PCR can be done to double-check correct insertion of the digested plasmid.

Thus, we can make an integrant using the Golden Gate method and modules presented in this study.

### 2.9. How to make GOI and new modules on demand

#### 2.9.1. Preparation of GOI modules

Next we introduce how to prepare the GOI modules. The GOI fragment must display cohesive ends at both ends. PCR is the best way to achieve this. A pair of oligonucleotides including the sequence of the *Bsa*I site (both recognition site and cohesive end) at the 5′-end must be prepared. For example, in the case of C-terminal FP tagging (figure 2*b*), forward and reverse primers must have ‘b’ and ‘c’ at their 5′-ends, respectively.

On the other hand, in the case of N-terminal FP tagging (figure 3*b*), forward and reverse primers should have ‘c’ and ‘d’, respectively. If you do not need any FPtag to fuse, the cohesive ends ‘b’ and ‘d’ should be added. Actual sequences of ‘b’, ‘c’ and ‘d’ are shown in figure 2*c* and table 2.

For easy oligonucleotide designing, we show a table summarizing which types of oligonucleotide primers you need (table 2). For instance, when you prepare a GOI (bc) module (figure 2), the forward oligonucleotide to amplify the GOI should be 5′-tttGGTCTCa**TATG**-(gene-specific)-3′, as shown in table 2. ‘ttt’ therein can be any other three nucleotides. GGTCTC is the recognition site for *Bsa*I, whereas the bold TATG becomes the cohesive end (b), which is exposed when the amplified double strand DNA is digested with *Bsa*I. The single base ‘a’ inserted in between serves as a spacer. The underlined ATG is expected to serve as the initiation codon (for methionine) of the GOI, as the TATG directly connects the 3′-end of the promoter module, which exposes the complementary cohesive end.

Similarly, table 2 indicates that the reverse oligonucleotide to amplify the GOI should be 5′-tttGGTCTCa**TCAG**-(gene-specific)-3′, where ‘ttt’ could be any other three bases, and the bold TCAG is the cohesive end (c) for the neighbouring FP module to be connected, which exposes the complementary cohesive end. Note that it is essential to not include termination codons at the end of the GOI in the oligonucleotide, when in-frame fusion with FPtag-C (cd) is planned.

The amplified products may need purification through gel electrophoresis and a standard DNA purification protocol. The purified product can be subjected to the Golden Gate reaction together with other modules.

#### 2.9.2. How to newly construct other modules

As we mentioned, we can use new modules that are not listed in table 1. Such new modules can be cloned in pCR-Blunt II-TOPO or other plasmids, which does not have the ampicillin resistance gene as a selection marker for *E. coli*, but cloning is not necessary. Instead, we recommend preparing linear DNA fragments with *Bsa*I sites attached to both ends using PCR as is the case for GOI, so that it can be directly applied to the Golden Gate reaction.

Information for oligonucleotide primer sequences necessary to construct new modules is summarized in table 2. For instance, to amplify a new promoter module through PCR, it is necessary to order a pair of oligonucleotide primers, sequences of which are shown in the rows of ‘I. Promoter (ab)’ (table 2). The primers amplify the promoter module fragment with cohesive ends ‘a’ and ‘b’ at 5′- and 3′-ends, respectively.

As mentioned earlier, a small epitope tag such as FLAG or HA can be introduced through PCR with an oligonucleotide primer that contains the tag sequence (see 2.6.IIIa and 2.7.VIIa for details). Alternatively, a DNA fragment for a small epitope tag can be prepared by annealing two complementary synthetic oligonucleotides, which are designed to expose the required overhang after annealing [30]. The fragment might be directly used as a module for the small epitope tag in the Golden Gate reaction. In this case, the oligonucleotides do not need to include *Bsa*I sites. This would further increase the flexibility of the system.

### 2.10. How target modules work

Additional explanation for target modules is given below. In this study, we chose the following four sites as target sites for integration: co2 and *lys1* on chromosome I, z2 on chromosome II and *arg1* on chromosome III.

Figure 4 illustrates the outline as to how we constructed target modules and the principle of how it works at integration, using the *lys1* target module as an example.

First, we chose an approximately 1 kb region in the middle of *lys1*^+^ gene as the target site, and named the first half of the region as ‘lys1F’, and the latter half as ‘lys1R’ (figure 4*a*). We amplified DNA fragments of both lys1F and lys1R, and the lys1R DNA fragment was placed ahead of the lys1F fragment, with an *Fse*I restriction site in between (figure 4*b*). Cohesive ends named ‘e’ and ‘f’ (figure 2*c* and table 2) were added at each end of the module and cloned in pCR-Blunt II-TOPO (figure 4*b*). Plasmids for other target modules presented in this study (figures 2 and 3; table 1) were constructed similarly. The resultant *lys1*^+^ target module as well as other necessary modules can be mixed to perform Golden Gate reaction, thereby producing an integration plasmid (figure 4*c*).

Inverted connection of the lys1F and lys1R sequences is not necessarily required for integration itself, as other integration plasmids invented previously are not in this style [6]. In those cases, however, integration of the linearized fragment would generate a repeat of an identical sequence at the head and the tail of the integration site on the chromosome [31,32]. This might increase the risk of spontaneous pop-out of the flanked region from the chromosome. In this study, we therefore inverted the order intentionally, which avoids production of repetitive sequences (figure 4*d*).

### 2.11. An example of integration using integration modules

As a proof of principle, we show examples of integrant strains made using the materials and methods presented in this study.

First, we created integration plasmids to express the fusion protein of Cut7 with GFP, with the P*nmt1* promoter (derived from #4), C-terminal GFP tag (#8), the *kan* selection marker (#20) and the co2 target sequence (#31). As a GOI, we used either of wild type and 10 truncated mutants of the *cut7* gene (1.2–3.0 kb). In total, 11 Golden Gate reactions were performed. Transformation of *E. coli* cells using each reaction sample was performed, and some colonies grown on LB + Amp were chosen for minipreps. Plasmids were then digested with *Not*I to confirm successful ligation. Rates for successful ligation were: 1/1 (in three reactions), 1/2 (in one reaction) and 3/3 (in seven reactions), demonstrating that the Golden Gate reaction using the materials efficiently yields correct plasmids.

**Figure 5.**
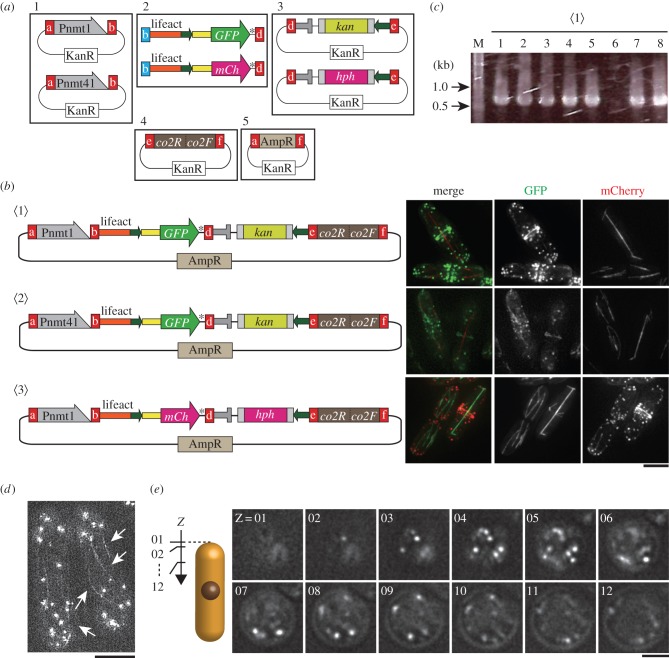
An application of the C-terminal tagging system. We constructed strains to visualize actin organization as a proof of principle. (*a*) Module plasmids used for Golden Gate reaction to make integration plasmids to express the actin-binding protein lifeact with GFP or mCherry. P*nmt1* (#4) or P*nmt41* (#5) module plasmids were chosen from the promoter module group I (1). The GOI module here (2) is the DNA fragment comprising the in-frame fusion gene of lifeact with GFP or mCherry. The fusion genes were made through PCR. Modules from 1 to 5 were mixed to perform a Golden Gate reaction. Modules are connected in the order shown here. (*b*) Standard fluorescence microscopy to confirm expression of lifeact-GFP and lifeact-mCherry. (left) Integration plasmids created through the Golden Gate method and used for transformation after linearization. 〈1〉, 〈2〉: plasmids for expression of lifeact-GFP under the *nmt1* 〈1〉 or *nmt41* 〈2〉 promoter. 〈3〉: a plasmid for expression of lifeact-mCherry under the *nmt1* promoter. All constructs were inserted at the co2 site of chromosome I. (right) Observation of lifeact-GFP and lifeact-mCherry together with microtubule markers mCherry-Atb2 and GFP-Atb2, respectively, in the strains created with the integration plasmids. Images are maximum intensity projections of deconvolved stacks. (*c*) Results of colony PCR to examine proper integration of the linearized plasmid 〈1〉. (*d*) Structural illumination microscopy to confirm cytoplasmic actin cables (arrows) as well as patches visualized with P*nmt1*-driven lifeact-GFP (the strain 〈1〉). Scale bars, 5 µm. The final image is a maximum-intensity projection of a 1 μm deep stack. (*e*) Head-on imaging of a cell expressing P*nmt1*-driven lifeact-GFP. Images were taken every 0.125 µm along the *z*-axis. Scale bar, 2 µm.

In the next example, we aim to visualize the actin cytoskeleton by the fusion gene of GFP or mCherry with lifeact [33,34], which is to be integrated in an *S. pombe* chromosome. Figure 5*a* is a schematic describing which modules were used. We prepared three reaction samples (figure 5*b*): 〈1〉 P*nmt1*-driven lifeact-GFP with the selection marker *kan*; 〈2〉 P*nmt41*-driven lifeact-GFP with *kan*; and 〈3〉 P*nmt1*-driven lifeact-mCherry with the selection marker *hph*. Five modules (1–5, figure 5*a*) were mixed for one Golden Gate reaction.

In these cases, the GOI should correspond to the lifeact gene, but it was too short in sequence (51 bp) to prepare through PCR. Instead, the lifeact sequence was included in the forward oligonucleotide primer to amplify the GFP (or mCherry) gene with the linker, using the module plasmid for the GFP tag (#8) (or mCherry tag, #9) as a template (2, figure 5*a*). At the same time, the forward and reverse primers carried cohesive ends ‘b’ and ‘d’ at the 5′ end, respectively. The amplified product (2) was the in-frame fusion gene of lifeact-GFP (or lifeact-mCherry) with the linker, so that it could be connected both to a promoter module (with ‘b’ in 1) and to a terminator + marker module (with ‘d’ in 3) (figure 5*a*). Researchers thus may modify any parts of Golden Gate modules according to the requirement.

After transformation of three Golden Gate samples, 30–150 transformed *E. coli* colonies were seen on LB + Amp plates. Eight colonies from each transformant were chosen for plasmid minipreps, and digestion with *Not*I confirmed that 5, 6 and 2 out of 8 samples showed correct ligation for the integration plasmid 〈1〉, 〈2〉 and 〈3〉, respectively. Correct integration plasmids were then digested with *Fse*I, and the linearized fragments were used for transformation of *S. pombe* cells expressing mCherry-tubulin or GFP-tubulin. Stable transformants grown in the selective medium were chosen for colony PCR to test whether accurate integration occurred. Integration was mostly accurate and efficient, as seven out of eight colonies for the strain 〈1〉 were positive (figure 5*c*). Positive colonies for each strain were cultured in rich medium overnight, and observed by microscopy. Actin patches, cables and rings could be visualized with lifeact-GFP and lifeact-mCherry in all three constructs by conventional widefield microscopy (figure 5*b*). The lifeact-GFP signal was also sufficiently robust to allow structured illumination microscopy (SIM), revealing individual actin cables in exquisite detail (figure 5*d*). With Pnmt1-driven lifeact-GFP actin patches could be viewed in vertical stacks taken of horizontally arrayed cells [3] (figure 5*e*).

These observations indicate that, at least in rich medium (YE5S) containing thiamine, Pnmt1 was better than Pnmt41 for visualization of the actin organization *in vivo*. Thus, the Golden Gate method works with the modules provided in this study, as expected.

## 3. Discussion

### 3.1. Tips for successful Golden Gate reaction

This study presents materials and methods that enable systematic construction of many kinds of integration vectors. This provides a flexible, scalable way to capitalize on the ever-increasing number of fluorescent protein and selection marker gene modules available for *S. pombe* work, a feature particularly important in the context of large-scale phenotyping projects [35–38].

For efficient Golden Gate reaction, we compiled some technical tips in experimental procedures.

The number of modules in a reaction does not matter for now: Golden Gate reaction works efficiently even with 5–8 DNA fragments of kilobase length. We have not tried reactions with more than nine modules of kilobase length, but Engler *et al*. [9] use 10 DNA fragments, albeit short, for one reaction. We therefore predict the number of modules can be increased even more, which will allow us to create plasmids containing more complicated gene constructs.

There are three main reasons for failure in the Golden Gate reaction in our experience. These points should be considered when the reaction does not work well.

First, decreased activity of *Bsa*I. *Bsa*I needs to be fully active for efficient Golden Gate reaction. We advise to consider using a new lot when in trouble. To test whether the current lot retains sufficient enzymatic activity, we recommend digestion of an existing module plasmid with *Bsa*I in the conditions for the Golden Gate reaction using a thermal cycler (figure 1*b*), rather than doing it simply at 37°C.

Second, concentration of each module was not equal, or was too high or too low. We strongly recommend to adjust the concentration of all modules to 20 fmol µl^−1^. Unequal concentration among modules significantly deteriorates the efficiency to produce circularized plasmids.

Third, unintended *Bsa*I sites existed in the GOI module. Examine whether the GOI (and other modules) contains unintended *Bsa*I sites inside. Existence of unintended *Bsa*I sites in modules blocks circularization of the integration plasmid. For this reason, we removed two internal *Bsa*I sites from pFA6a to create the vector module (#35, table 1), and one site from GFP(S65 T) to make modules with the GFP tag (#8, #11, #14 and #17).

When your GOI contains internal *Bsa*I sites, we suggest two solutions. One way is to clone the GOI and to remove the sites through site-directed mutagenesis. Alternatively, the GOI can be prepared as multiple modules. For example, if a GOI contains two *Bsa*I sites in the middle, split it into three parts at the position of the *Bsa*I sites (e.g. fragments P, Q and R). Each portion can be amplified through PCR separately with oligonucleotide primers with a particular combination of cohesive ends, to yield three GOI fragments: b-P-x, x-Q-y and y-R-c. Note that ‘b’ and ‘c’ are cohesive ends connectable to the promoter and FPtag modules (figure 2 and table 1), whereas ‘x’ and ‘y’ are not-yet-used cohesive ends. Four-base sequences of ‘x’ and ‘y’ can be determined arbitrarily, but they need to be different from those of ‘a’–‘g’, which have been already used in this study, and also from ‘GTTA’, the cohesive end generated from the pBMod backbone. Importantly, the oligonucleotide primers need to be designed to remove the recognition sequence (5′-GGTCTC-3′) of the original internal *Bsa*I site, by a silent mutation. As the number of DNA fragments for a Golden Gate reaction is not yet saturated, we can increase the number of GOIs to solve this issue.

### 3.2. Many kinds of plasmids can be systematically made

As shown in figure 5, it is difficult to predict how much of an external gene product (lifeact in that case) should be expressed in *S. pombe* cells. We then need to make several kinds of integration plasmids to try several promoters with distinct activity (P*nmt1* and P*nmt41* in figure 5).

Alternatively, we may want to prepare both GFP-fusion and mCherry-fusion strains (lifeact-GFP and lifeact-mCherry in figure 5). But it might be rare to find existing ready-made integration plasmids that already contain all the modules we wish for.

Plasmid construction using the Golden Gate DNA shuffling method is powerful in those cases. A number of modules that we normally use are presented in this study, and in many cases all that you need may be just a GOI module. Once the GOI module is prepared through a simple PCR reaction, the GOI module can be mixed in combination with many kinds of modules as you wish. Any combination of modules can be systematically constructed in a single step in a single tube. Theoretically, the methods can be applied also for other purposes than integration; therefore, we expect the system presented in this study may represent a practical break-through in plasmid construction.

## 4. Material and methods

### 4.1. Fission yeast genetics, strains and media

Strains used in this study are listed in table 3. Standard methods were used for *S. pombe* genetics [39,40]. For growth of strains, the rich medium YE5S (yeast extract based medium with adenine, uracil, leucine, lysine and histidine as supplements) was used. A standard protocol for *S. pombe* transformation using lithium acetate was used [12]. According to the protocol, cells after transformation were placed on the YE5S agar plates overnight. Those were then replica-plated onto YE5S plates containing G418 (100 µg ml^−1^, Roche, Basel or Wako, Osaka), hygromycin B (100 µg ml^−1^, Wako, Osaka), clonNAT (100 µg ml^−1^, WERNER BioAgents, Jena) or blasticidin S (300 µg ml^−1^, Funakoshi, Tokyo), for selection of transformants by *kan*, *hph, nat* or *bsd*, respectively [12,24].

**Table 3.** *S. pombe* strains used in this study. *leu1*, *leu1-32*; *ura4*, *ura4-D18*.

strain	genotype	origin	integration plasmid used	figure
MJ1100	h90 Z2-GFP-atb2-kan leu1 ura4 ade6-M216	this study	—	5*b*
MJ1105	h90 Z2-mCherry-atb2-hph leu1 ura4 ade6-M216	this study	—	5*b*
TOM0165	h90 Z2-mCherry-atb2-hph co2::Pnmt1-lifeact-GFP-Tadh-kan leu1 ura4 ade6-M216	this study	pFA-Pnmt1-lifeact-GFP-Tadh-kan-CO2 #1^a^	5*b*–*e*
TOM0167	h90 Z2-mCherry-atb2-hph co2::Pnmt1-lifeact-GFP-Tadh-kan leu1 ura4 ade6-M216	this study	pFA-Pnmt1-lifeact-GFP-Tadh-kan-CO2 #2^a^	5*b*,*c*
TOM0177	h90 Z2-mCherry-atb2-hph co2::Pnmt41-lifeact-GFP-Tadh-kan leu1 ura4 ade6-M216	this study	pFA-Pnmt41-lifeact-GFP-Tadh-kan-CO2	5*b*
TOM0184	h90 Z2-GFP-atb2-kan co2::Pnmt1-lifeact-mCherry-Tadh-hph leu1 ura4 ade6-M216	this study	pFA-Pnmt1-lifeact-mCherry-Tadh-hph-CO2	5*b*

^a^Plasmids #1 and #2 are two independent clones obtained from a single Golden Gate reaction.

For confirmation of proper integration at the co2 site of chromosome I, colony PCR was performed with a pair of oligonucleotide primers: co2CP1F: 5′-AAGCCTCGTCTAAGCGAATC-3′ and Ttef-NtoC: 5′-CGACATCATCTGCCCAGATGCG-3′.

### 4.2. Golden Gate DNA shuffling reaction

Our Golden Gate DNA shuffling method is based on the original report of the method [9,10]. Detailed experimental procedures are described in the Results section. Sequences of cohesive ends a–g (figure 2*c*; tables 1 and 2) were defined in this study.

### 4.3. Construction of modules

Modules except for GOI (N) and GOI (C) were cloned into the plasmid pCR-Blunt II-TOPO after PCR amplification, according to instruction given in the Zero Blunt TOPO PCR cloning kit (#K2800-20; Invitrogen, MI, USA). The sequence of a *Bsa*I recognition site and a designed cohesive end was added to each side of module using a pair of oligonucleotide primers to amplify the module fragments.

Origin of module fragments is as follows: for Promoter modules, the *nmt1* promoter P*nmt1* [15] was amplified from *S. pombe* genomic DNA (chromosome III: 1 837 263–1 838 542) in pombase [41]. Its derivatives P*nmt41* and P*nmt81* were made through site-directed mutagenesis using the PrimeSTAR mutagenesis kit (#R046A; Takara Bio, Ohtsu). The *adh1* promoter P*adh1* and its derivatives P*adh41* and P*adh81* were prepared similarly (chromosome III: 1 590 560–1 591 304).

For fluorescent protein modules, coding sequences for *Aequorea victoria* GFP (S65 T) and its variant CFP (ECFP) were amplified, using pFA6a-GFP(S65 T)-kanMX [12] and pFA6a-ECFP-nat [18] as a template. The mCherry gene originally derived from *Discosoma* sp. was amplified similarly using pFA6a-mCherry-hphMX [18] as a template.

Modules containing the *adh1* terminator (with or without a selection marker) were amplified from pFA6a-based plasmids containing antibiotics resistance genes *kan* [21], *hph* and *nat* [24,25], *bsd* [19], as well as uracil and leucine autotroph markers *ura4*^+^ and *LEU2*, respectively.

The vector module was derived from the plasmid pFA6a [21]. The original pFA6a, however, contains two internal *Bsa*I sites, which hampers its use as an entry plasmid for the Golden Gate system. Therefore, we removed the sites as follows: a short fragment comprising 137 bases (5′-CAGCTGAAGC-· · ·-TGCCGGTCTC-3′), which contains a *Bsa*I recognition site, was removed from the original pFA6a, and the other *Bsa*I recognition site in the ampicillin resistance gene was eliminated through site-directed mutagenesis.

Target modules were created as follows. Regions corresponding to 2 939 891–2 940 410 (co2F) and 2 939 404–2 939 887 (co2R) of chromosome I were amplified through PCR. These two were connected in the inverted order and an additional *Fse*I site was inserted at the border, which yields the fragment ‘co2R-*Fse*I-co2F’.

Similarly, z2R (3 485 793–3 486 320 of chromosome II) and z2F (3 485 246–3 485 782) were connected to make the fragment ‘z2R-*Fse*I-z2F’. The *arg1* module (arg1R-*Fse*I-arg1F) is composed of arg1R (1 614 151–1 614 549 of chromosome III) and arg1F (1 613 754–1 614 150) connected with an *Fse*I site. The lys1 module (lys1R-*Fse*I-lys1F) is composed of lys1R (3 740 972–3 741 481 of chromosome I) and lys1F (3 740 472–3 740 971) connected with an *Fse*I site.

### 4.4. Modules to visualize actin

Fusion genes of lifeact-GFP and lifeact-mCherry were made as follows. The gene encoding GFP(S65T) with the linker sequence was amplified using pBMod-GFP(C) (#8) as a template with a pair of oligonucleotide primers: LA-linker-GG-F-2: 5′-tttGGTCTCaTATGGGTGTCGCTGACCTTATCAAGAAGTTCGAGTCTATTTCTAAGGAAGAAGGGATCCCCGGGTTAATTAAGG-3′ and GFP-R: 5′-tttGGTCTCaAGACTTAACTAGTTTTGTATAGTTCATCCATGCC-3′. The underlined sequence encodes the lifeact protein [33]. For lifeact-mCherry, LA-linker-GG-F2 and mCherry-R: 5′-tttGGTCTCaAGACTTAACTAGTCTTGTACAGCTCGTCCATGC-3′ were used.

### 4.5. Microscopy

Cells were grown for 12 h in YE5S liquid medium and then subjected to microscopy. The DeltaVision SoftWoRx system was used for conventional microscopy, as described previously [18]. Briefly, cells were mounted on a lectin-coated glass-bottomed dish, which was then filled with the Edinburgh Minimal Medium (EMM) with five supplements (as in YE5S). Ten sectional images were acquired along the *z*-axis, with 0.4 µm intervals.

Methods for head-on cell imaging were performed as described previously [3]. In brief, prior to imaging, all cells were grown at 32°C to exponential in YE5S liquid media and then mounted on lectin-coated glass-bottomed dishes, and conventional widefield microscopy of vertically arrayed cells was performed with an OMX microscope (Applied Precision) in conventional mode. For horizontally arrayed cells, structured illumination microscopy was performed with an OMX microscope in live-cell structured illumination mode, and stacks of 1 μm depth with planes 0.125 μm were taken and processed in SoftWoRx.

## Supplementary Material

Table 1. Module plasmids created in this study

## Supplementary Material

Table 2. Guideline for designing oligonucleotide primers with cohesive ends

## Data accessibility

All relevant data are within the paper.
